# Supplementary material for: Integrated Analysis of Ginsenoside Content and Biomarker Changes in Processed Ginseng: Implications for Anti-Cancer Mechanisms
Source: Foods. 2024 Aug 8;13(16):2497. doi: 10.3390/foods13162497 (PMC11353654; doi:10.3390/foods13162497)

Supporting Information

Table S1 Standards Information.

No.	Type	Name	S/N
1	Prototype Ginsenoside	Ginsenoside Rb1（Rb1）	DSTDR000601
2		Ginsenoside Rb2	DSTDR000701
3		Ginsenoside Rb3	DST200315-008
4		Ginsenoside Rc	DST191110-013
5		Ginsenoside Rd	DST200703-015
6		Ginsenoside Re	DSTDR001401
7		Ginsenoside Rf	DST200919-017
8		Ginsenoside Rg1	DST200722-009
9		Ginsenoside Ro	DSTDR003101
10		Ginsenoside F2	DST200524-026
11		Ginsenoside F3	DST200621-027
12	Rare Ginsenoside	Ginsenoside S-Rg2	DSTDR001001
13		Ginsenoside S-Rg3	DST191107-011
14		Ginsenoside Rg5	DST210922-032
15		Ginsenoside R-Rh1	DST190922-018
16		Ginsenoside S-Rh2	DSTDS003601
17		Ginsenoside Rk1	DST200409-034
18		Ginsenoside Rk3	DST220218-036
19		Ginsenoside F4	DST200302-015
20		Ginsenoside CK	DST200409-030

Table S2 MRM parameters for the quantitation of ginsenosides.

Compound	t_R/min	Ion Mode	Precursor Ion（*m/z*）	Q1 Pre /V	Collision Energy /V	Q3 Pre/V
Rb1	8.668	+	1131.9000>365.1000	-32	-55	-25
Rb2	10.534	-	1077.4500>191.1500	32	54	19
Rb3	10.838	-	1077.5000>149.1000	34	55	15
Rc	9.559	-	1077.5000>148.9500	32	55	10
Rd	12.238	-	945.4000>101.0000	24	51	18
Re	1.343	-	945.4000>783.3500	38	38	30
Rf	6.097	-	799.4500>475.2500	24	43	24
Rg1	1.383	-	845.5000>637.3500	26	34	24
S-Rg2	8.485	-	783.3500>475.2500	32	42	24
Ro	10.066	-	955.5000>793.2500	22	49	28
S-Rg3	16.302	-	783.5000>100.9500	24	46	10
Rg5	17.347	-	765.4000>70.9500	24	46	26
R-Rh1	8.452	-	683.5000>44.9500	26	27	16
S-Rh2	17.530	-	667.5000>44.8500	24	23	15
Rk1	17.178	-	765.4500>603.2000	22	31	30
RK3	15.895	-	665.2500>44.9000	34	25	17
F2	15.869	-	829.4500>783.4500	26	22	30
F3	8.402	-	769.5000>148.9500	24	31	10
F4	15.839	-	811.3500>765.4000	24	21	30
CK	17.288	-	667.4500>621.3000	20	20	24

Table S3 The result of method validation.

Compound	Linearity	*r*	Range /(μg·mL^{-1})	LOD /(μg·mL^{-1})	LOQ /(μg·mL^{-1})	RSD/%			Recovery	
						Precision	Repeatability	Stability	mean/%	RSD/%
Rb1	Y = 15187.7X	0.9995	0.5-40	0.078	0.622	2.19	2.85	2.82	103.65	3.58
Rb2	Y = 17069.0X	0.9990	0.05-50	0.012	0.079	2.93	2.40	1.94	106.08	7.12
Rb3	Y = 74763.2X	0.9999	0.05-20	0.032	0.219	2.05	0.50	2.30	102.39	6.88
Rc	Y = 23553.8X	0.9998	0.05-60	0.037	0.163	2.91	2.11	1.34	96.74	2.50
Rd	Y = 15255.0X	0.9992	0.05-100	0.389	3.357	2.64	2.49	1.85	96.50	2.01
Re	Y = 2354.07X	0.9998	0.05-200	0.975	7.482	2.84	1.86	3.88	104.56	5.79
Rf	Y = 83908.2X	0.9999	0.1-20	0.183	1.094	2.50	2.77	1.78	102.37	6.19
Rg1	Y = 58063.2X	0.9999	0.05-100	0.253	0.601	2.42	1.40	3.81	98.32	3.89
S-Rg2	Y = 23015.2X	0.9993	0.1-50	0.004	0.244	2.17	2.03	4.09	92.42	6.78
Ro	Y = 17315.7X	0.9999	0.1-100	0.002	0.018	2.91	2.87	2.67	104.27	3.16
S-Rg3	Y = 104242X	0.9993	0.001-50	0.08	0.821	2.92	1.31	2.89	96.65	2.27
Rg5	Y = 87317.6X	0.9994	0.001-50	0.001	0.013	1.82	1.70	4.04	98.32	2.38
R-Rh1	Y = 116737X	0.9998	0.05-20	0.02	0.144	2.50	1.64	4.25	106.27	4.36
S-Rh2	Y = 243622X	0.9992	0.001-50	0.001	0.015	2.73	1.36	3.34	97.64	4.37
Rk1	Y = 4466.32X	0.9995	0.05-200	0.015	0.117	2.33	2.61	2.82	102.30	3.60
RK3	Y = 178346X	0.9993	0.001-20	0.02	0.156	2.52	1.81	4.04	95.09	5.97
F2	Y = 101357X	0.9996	0.05-150	1.827	14.412	2.75	2.73	3.98	96.95	3.03
F3	Y = 21635.3X	0.9997	0.05-50	0.004	0.028	1.78	1.38	3.70	87.78	5.81
F4	Y = 545211X	0.9995	0.001-150	0.02	0.159	2.69	2.51	5.71	100.27	5.35
CK	Y = 286848X	0.999	0.001-20	0.002	0.084	1.32	1.94	3.01	97.86	2.44

Compared to the WG group: *p<0.05, **p<0.01

Table S4 The quantitative results of all samples.

		Rb1		Rb2		Rb3		Rc	
		WG	BG	WG	BG	WG	BG	WG	BG
	YS	3.923±3.461	0.84±0.768	7.187±1.33	4.404±0.873**	0.741±0.099	0.409±0.105**	7.089±1.205	3.601±0.677**
Leaf	LXS-1	0.456±0.454	1.471±0.774	2.149±0.776	3.069±0.764	0.189±0.046	0.236±0.035	1.832±0.733	2.039±0.576
	LXS-2	0.643±0.687	1.243±0.827	3.671±1.942	3.964±0.771	0.337±0.146	0.383±0.076	3.532±1.527	3.098±0.706
	YS	0.001±0.001	0.002±0.001	0.04±0.018	0.006±0.005*	0.016±0.006	0.001±0.002**	0.011±0.01	0.003±0.002
Stem	LXS-1	0.006±0.007	0.001±0.002	0.029±0.023	0.01±0.017	0.009±0.006	0.002±0.005	0.013±0.012	0.003±0.005
	LXS-2	0.005±0.006	0.006±0.012	0.058±0.024	0.033±0.014	0.015±0.005	0.008±0.005	0.022±0.007	0.007±0.004**
	YS	34.793±10.548	17.047±15.308	1.465±0.514	1.312±0.858	0.21±0.077	0.175±0.095	2.122±0.715	1.35±0.891
Rhizome	LXS-1	74.6±12.116	35.616±8.564**	3.417±1.292	1.904±0.812	0.636±0.279	0.26±0.098	4.853±1.79	2.098±0.761*
	LXS-2	83.051±18.851	79.285±9.9	2.923±1.574	3.852±0.977	0.62±0.375	0.637±0.164	4.568±2.156	4.897±1.078
	YS	35.251±14.658	1.258±2.695*	1.707±1.122	0.242±0.192	0.26±0.157	0.033±0.031	2.651±1.364	0.24±0.187
Main root	LXS-1	71.072±20.747	0.308±0.495**	2.373±0.995	0.074±0.096*	0.444±0.166	0.012±0.013*	3.529±1.005	0.103±0.131**
	LXS-2	73.632±32.341	6.261±2.983	1.524±1.288	0.25±0.056	0.325±0.227	0.05±0.01	2.441±1.6	0.404±0.07
	YS	82.249±24.836	78.273±10.552	5.215±3.717	5.994±2.065	1.133±0.823	1.027±0.225	9.641±5.056	7.927±1.265
Lateral root	LXS-1	106.896±23.867	57.813±13.323*	5.85±3.403	2.445±1.873	1.251±0.665	0.434±0.241	8.469±3.671	3.236±1.449
	LXS-2	118.935±55.477	112.127±10.21	3.281±2.862	3.296±1.581	1.06±0.859	0.784±0.226	6.767±4.225	5.628±1.238

Compared to the WG group: *p<0.05, **p<0.01

		Rd		Re		Rf		Rg1	
		WG	BG	WG	BG	WG	BG	WG	BG
Leaf	YS	21.025±4.098	15.914±2.127	41.699±4.801	16.334±2.643**	0.288±0.087	0.184±0.045	8.673±1.652	2.927±0.396**
	LXS-1	10.056±2.802	17.062±2.928**	42.693±7.994	21.873±7.946**	0.266±0.072	0.287±0.081	5.282±1.403	3.136±0.953
	LXS-2	11.991±3.102	17.069±2.568	26.068±3.083	10.735±2.478**	0.24±0.085	0.256±0.03	8.56±2.065	3.702±0.52**
Stem	YS	0.146±0.038	0.141±0.038	3.881±0.877	0.133±0.314**	0.281±0.035	0.145±0.019**	3.595±0.892	0.087±0.134**
	LXS-1	0.269±0.197	0.245±0.099	4.051±0.623	0.116±0.108**	0.302±0.035	0.128±0.031**	2.45±0.525	0.106±0.077**
	LXS-2	0.136±0.041	0.588±0.254	3.836±0.859	0.235±0.141**	0.34±0.037	0.237±0.045**	2.948±0.502	0.225±0.101**
Rhizome	YS	0.516±0.191	1.511±0.472**	7.629±1.524	0.251±0.233**	1.389±0.279	1.263±0.295	5.437±0.987	0.18±0.127**
	LXS-1	0.836±0.196	1.553±0.512*	11.13±2.218	0.491±0.139**	2.184±0.406	1.493±0.201	8.752±1.962	0.605±0.186**
	LXS-2	0.834±0.37	2.331±0.305**	8.246±1.403	1.774±0.356**	2.285±0.759	2.308±0.191	12.486±3.436	3.983±0.911*
Main root	YS	0.573±0.282	0.41±0.391	5.977±1.915	0.027±0.053**	1.679±0.679	0.963±0.599	8.984±3.698	0.039±0.048*
	LXS-1	0.724±0.296	0.101±0.073*	5.927±0.743	0.01±0.024**	1.655±0.469	0.551±0.275*	12.341±4.469	0.028±0.018*
	LXS-2	0.878±0.73	0.342±0.102	4.638±1.154	0.097±0.063**	1.453±0.731	1.05±0.2	11.271±4.679	0.18±0.055*
Lateral root	YS	3.283±1.149	7.369±0.969**	15.23±3.214	1.565±0.531**	2.206±0.414	1.864±0.315	4.52±0.802	0.511±0.152**
	LXS-1	2.321±0.689	3.329±0.458	12.878±2.545	1.277±0.419**	1.968±0.557	1.497±0.387	7.066±2.533	0.584±0.167*
	LXS-2	2.435±1.154	4.597±1.011**	13.658±2.934	3.14±0.971**	2.356±0.575	2.378±0.255	7.918±1.393	2.257±0.436**

Compared to the WG group: *p<0.05, **p<0.01

		S-Rg2		Ro		S-Rg3		Rg5	
		WG	BG	WG	BG	WG	BG	WG	BG
Leaf	YS	2.77±0.415	13.345±0.824**	0.626±0.275	0.449±0.223	0.231±0.048	4.718±0.565**	0.001±0	3.723±0.467**
	LXS-1	2.576±0.663	11.222±1.363**	0.795±0.318	0.721±0.417	0.095±0.025	3.797±0.473**	0.001±0.001	2.859±0.428**
	LXS-2	1.562±0.205	10.225±0.827**	0.404±0.239	0.541±0.329	0.245±0.054	5.289±0.366**	0.003±0.001	4.865±0.442**
Stem	YS	0.544±0.139	1.212±0.241**	1.029±0.263	0.554±0.23*	0.001±0	0.531±0.109**	0	0.443±0.115**
	LXS-1	0.624±0.101	0.88±0.096	0.623±0.227	0.376±0.18	0.002±0.001	0.332±0.051**	0	0.357±0.068**
	LXS-2	0.496±0.127	1.246±0.236**	0.74±0.272	0.542±0.158	0.001±0.001	0.541±0.267	0	0.499±0.241
Rhizome	YS	0.719±0.263	2.388±0.225**	11.566±2.873	8.379±1.75	0.005±0.002	5.761±0.932**	0.003±0.001	6.602±0.991**
	LXS-1	0.997±0.319	2.014±0.378**	14.65±2.525	10.323±1.838*	0.008±0.003	5.317±0.835**	0.006±0.004	6.586±0.975**
	LXS-2	0.588±0.082	2.096±0.269**	14.717±2.579	11.09±0.83	0.018±0.018	6.51±0.429**	0.008±0.004	7.119±0.671**
Main root	YS	0.55±0.272	1.34±0.611	8.91±3.329	4.691±0.503	0.009±0.004	5.017±1.412**	0.003±0.002	6.97±1.77**
	LXS-1	0.583±0.04	0.598±0.147	7.018±1.158	4.142±1.733	0.01±0.004	3.605±0.928**	0.006±0.004	5.316±1.195**
	LXS-2	0.354±0.172	1.024±0.24*	6.155±1.842	3.97±0.521	0.008±0.003	4.595±0.867**	0.008±0.004	5.871±1.127**
Lateral root	YS	2.345±0.894	5.957±1.071**	7.178±2.261	4.926±1.275	0.032±0.014	9.597±0.702**	0.004±0.001	9.606±0.939**
	LXS-1	1.659±0.512	2.917±0.565	4.983±1.033	4.612±1.818	0.025±0.008	7.539±0.928**	0.006±0.005	9.326±0.925**
	LXS-2	1.415±0.452	3.418±0.584**	4.393±1.593	3.54±0.623	0.026±0.012	8.411±0.789**	0.009±0.005	8.896±0.795**

Compared to the WG group: *p<0.05, **p<0.01

		R-Rh1		S-Rh2		Rk1		RK3	
		WG	BG	WG	BG	WG	BG	WG	BG
Leaf	YS	0.178±0.064	2.643±0.492**	0.017±0.012	2.154±0.461**	0.006±0.011	16.548±2.237**	0.001±0	1.429±0.254**
	LXS-1	0.075±0.012	1.571±0.141**	0.037±0.023	1.61±0.438**	0.001±0.002	13.481±1.866**	0	1.025±0.133**
	LXS-2	0.157±0.054	3.508±0.511**	0.092±0.061	3.814±1.036**	0.01±0.007	20.699±1.898**	0.001±0.001	1.98±0.222**
Stem	YS	0.019±0.005	0.739±0.049**	0	0.125±0.047*	0.003±0.006	2.684±0.509**	0	0.529±0.062**
	LXS-1	0.016±0.007	0.583±0.069**	0.001±0	0.103±0.052	0	2.045±0.388**	0	0.547±0.071**
	LXS-2	0.018±0.004	0.875±0.184**	0.001±0.001	0.114±0.04*	0	2.451±0.904*	0	0.691±0.126**
Rhizome	YS	0.029±0.009	2.626±0.418**	0.001±0.002	0.483±0.109**	0.002±0.003	30.438±4.829**	0	1.759±0.358**
	LXS-1	0.029±0.012	2.917±0.738**	0.001±0	0.277±0.06**	0.026±0.012	31.016±4.641**	0.001±0.001	2.129±0.487**
	LXS-2	0.03±0.013	3.343±0.234**	0.004±0.006	0.292±0.045**	0.035±0.026	38.23±1.01**	0.001±0.001	2.45±0.131**
Main root	YS	0.051±0.026	3.051±1.542	0	0.355±0.168	0.026±0.024	29.171±6.906**	0.002±0.002	2.357±0.912*
	LXS-1	0.076±0.052	2.085±0.793*	0	0.182±0.07*	0.035±0.024	24.207±5.221**	0.004±0.003	1.923±0.689*
	LXS-2	0.067±0.049	3.923±0.659**	0	0.136±0.065	0.029±0.016	28.742±5.221**	0.004±0.002	3.228±0.338**
Lateral root	YS	0.042±0.01	2.468±0.42**	0.001±0.001	0.841±0.131**	0.013±0.016	48.456±4.903**	0.001±0	1.478±0.241**
	LXS-1	0.046±0.031	2.686±0.434**	0.002±0.001	0.5±0.082**	0.019±0.014	44.386±3.391**	0	2.061±0.305**
	LXS-2	0.029±0.011	3.466±0.751**	0.002±0.001	0.562±0.118**	0.027±0.019	45.831±4.331**	0.001±0	2.174±0.367**

Compared to the WG group: *$p<0.05$, **$p<0.01$

		F2		F3		F4		CK	
		WG	BG	WG	BG	WG	BG	WG	BG
Leaf	YS	6.339±3.588	6.094±1.281	14.892±2.214	4.935±0.69**	0.024±0.006	15.903±0.834**	0.013±0.003	0.622±0.073**
	LXS-1	11.254±5.507	7.594±2.448	9.918±1.973	4.183±0.649**	0.011±0.004	14.342±2.293**	0.052±0.044	0.751±0.124**
	LXS-2	15.312±5.211	8.482±1.971	8.563±1.149	2.774±0.441**	0.026±0.007	20.848±0.782**	0.05±0.027	1.18±0.154**
Stem	YS	0.314±0.139	0.199±0.264	0.412±0.103	0.025±0.044**	0.005±0.003	3.698±0.517**	0.001±0	0.024±0.01*
	LXS-1	0.355±0.131	0.154±0.102	0.245±0.093	0.015±0.01*	0.002±0	3.699±0.395**	0.003±0.001	0.023±0.009*
	LXS-2	0.254±0.091	0.28±0.144	0.334±0.116	0.044±0.008*	0.004±0.002	3.83±0.409**	0.001±0.001	0.046±0.017*
Rhizome	YS	0.023±0.012	0.082±0.034	0.011±0.013	0.033±0.057	0.009±0.003	7.343±0.605**	0.011±0.008	0.039±0.006**
	LXS-1	0.025±0.01	0.06±0.02	0.005±0.004	0.02±0.025	0.015±0.005	6.439±1.09**	0.021±0.014	0.03±0.007
	LXS-2	0.072±0.071	0.148±0.046	0.009±0.009	0.014±0.016	0.015±0.005	6.106±1.137**	0.05±0.046	0.049±0.012
Main root	YS	0.015±0.012	0.086±0.071	0.001±0.003	0.031±0.054	0.01±0.003	5.816±2.071*	0.012±0.01	0.012±0.01
	LXS-1	0.008±0.003	0.049±0.015*	0.002±0.002	0.017±0.019	0.01±0.002	3.312±0.635**	0.007±0.003	0.004±0.002
	LXS-2	0.01±0.006	0.058±0.008**	0.009±0.012	0.018±0.031	0.011±0.004	4.336±0.979**	0.008±0.005	0.007±0.003
Lateral root	YS	0.056±0.026	0.35±0.073**	0.01±0.007	0.012±0.011	0.018±0.009	13.429±2.054**	0.078±0.067	0.148±0.035
	LXS-1	0.074±0.036	0.25±0.044**	0.013±0.018	0.023±0.022	0.015±0.005	8.902±0.907**	0.044±0.027	0.071±0.012
	LXS-2	0.092±0.039	0.413±0.198	0.018±0.01	0.035±0.052	0.021±0.011	8.024±1.219**	0.056±0.045	0.096±0.029

Compared to the WG group: *p<0.05, **p<0.01

		Total ginsenoside content	
		WG	BG
Leaf	YS	115.724±14.844	117.176±8.782
	LXS-1	87.738±12.753	112.328±17.66
	LXS-2	81.467±7.15	124.656±7.52
Stem	YS	10.3±1.712	11.28±1.524
	LXS-1	9±0.602	9.725±1.151
	LXS-2	9.21±1.112	12.497±2.506
Rhizome	YS	65.938±15.947	89.024±21.183
	LXS-1	122.191±18.45	111.149±16.55
	LXS-2	130.561±29.89	176.514±12.381
Main root	YS	66.672±25.661	62.11±18.349
	LXS-1	105.822±27.805	46.624±10.368
	LXS-2	102.824±42.852	64.54±11.953
Lateral root	YS	133.254±39.518	201.796±17.141
	LXS-1	153.586±34.969	153.89±22.498
	LXS-2	162.499±67	219.073±9.342

Table S5 The trends of ginsenoside levels after processing in each part.

		Rb1	Rb2	Rb3	Rc	Rd	Re	Rf	Rg1	S-Rg2	Ro	S-Rg3	Rg5	R-Rh1	S-Rh2	Rk1	RK3	F2	F3	F4	CK
Leaf	YS	↓	↓**	↓**	↓**	↓	↓**	↓	↓**	↑**	↓	↑**	↑**	↑**	↑**	↑**	↑**	↓	↓**	↑**	↑**
	LXS-1	↑	↑	↑	↑	↑**	↓**	↑	↓	↑**	↓	↑**	↑**	↑**	↑**	↑**	↑**	↓	↓**	↑**	↑**
	LXS-2	↑	↑	↑	↓	↑	↓**	↑	↓**	↑**	↑	↑**	↑**	↑**	↑**	↑**	↑**	↓	↓**	↑**	↑**
Stem	YS	↑	↓*	↓**	↓	↓	↓**	↓**	↓**	↑**	↓*	↑**	↑**	↑**	↑*	↑**	↑**	↓	↓**	↑**	↑*
	LXS-1	↓	↓	↓	↓	↓	↓**	↓**	↓**	↑	↓	↑**	↑**	↑**	↑	↑**	↑**	↓	↓*	↑**	↑*
	LXS-2	↑	↓	↓	↓**	↑	↓**	↓**	↓**	↑**	↓	↑	↑	↑**	↑*	↑*	↑**	↑	↓*	↑**	↑*
Rhizome	YS	↓	↓	↓	↓	↑**	↓**	↓	↓**	↑**	↓	↑**	↑**	↑**	↑**	↑**	↑**	↑	↑	↑**	↑**
	LXS-1	↓**	↓	↓	↓*	↑*	↓**	↓	↓**	↑**	↓*	↑**	↑**	↑**	↑**	↑**	↑**	↑	↑	↑**	↑
	LXS-2	↓	↑	↑	↑	↑**	↓**	↑	↓*	↑**	↓	↑**	↑**	↑**	↑**	↑**	↑**	↑	↑	↑**	↓
Main root	YS	↓*	↓	↓	↓	↓	↓**	↓	↓*	↑	↓	↑**	↑**	↑	↑	↑**	↑*	↑	↑	↑*	↓
	LXS-1	↓**	↓*	↓*	↓**	↓*	↓**	↓*	↓*	↑	↓	↑**	↑**	↑*	↑*	↑**	↑*	↑*	↑	↑**	↓
	LXS-2	↓	↓	↓	↓	↓	↓**	↓	↓*	↑*	↓	↑**	↑**	↑**	↑	↑**	↑**	↑**	↑	↑**	↓
Lateral root	YS	↓	↑	↓	↓	↑**	↓**	↓	↓**	↑**	↓	↑**	↑**	↑**	↑**	↑**	↑**	↑**	↑	↑**	↑
	LXS-1	↓*	↓	↓	↓	↑	↓**	↓	↓*	↑	↓	↑**	↑**	↑**	↑**	↑**	↑**	↑**	↑	↑**	↑
	LXS-2	↓	↑	↓	↓	↑**	↓**	↑	↓**	↑**	↓	↑**	↑**	↑**	↑**	↑**	↑**	↑	↑	↑**	↑

Compared to the WG group: *p<0.05, **p<0.01. ↓ Compared to WG, the content decreases, ↑ compared to WG, the content increases

Table S6 Percentage change in Rb1, Re, Rg1 of Ginseng Before and After Processing

		Rb1	Re	Rg1
Leaf	YS	-78.590	-60.828	-66.253
	LXS-1	222.547	-48.768	-40.631
	LXS-2	93.121	-58.819	-56.748
Stem	YS	66.102	-96.584	-97.568
	LXS-1	-82.124	-97.139	-95.666
	LXS-2	36.840	-93.866	-92.363
Rhizome	YS	-51.005	-96.705	-96.681
	LXS-1	-52.258	-95.587	-93.090
	LXS-2	-4.534	-78.484	-68.101
Main root	YS	-96.431	-99.550	-99.569
	LXS-1	-99.566	-99.835	-99.772
	LXS-2	-91.497	-97.908	-98.401
Lateral root	YS	-4.834	-89.727	-88.704
	LXS-1	-45.917	-90.085	-91.730
	LXS-2	-5.724	-77.010	-71.499

Figure S1 Comparison of ginsenoside content in five parts of cultivated ginseng before and after processing.

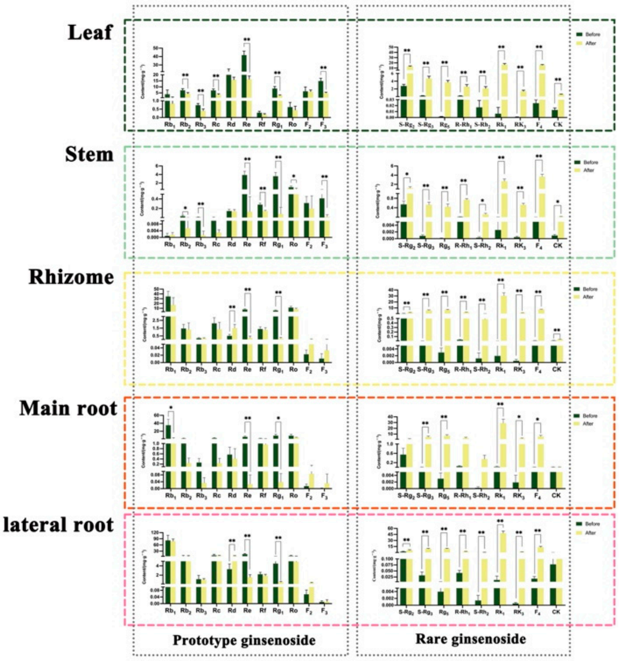

Figure S2 Comparison of ginsenoside content in five parts of 15-year forest ginseng before and after processing.

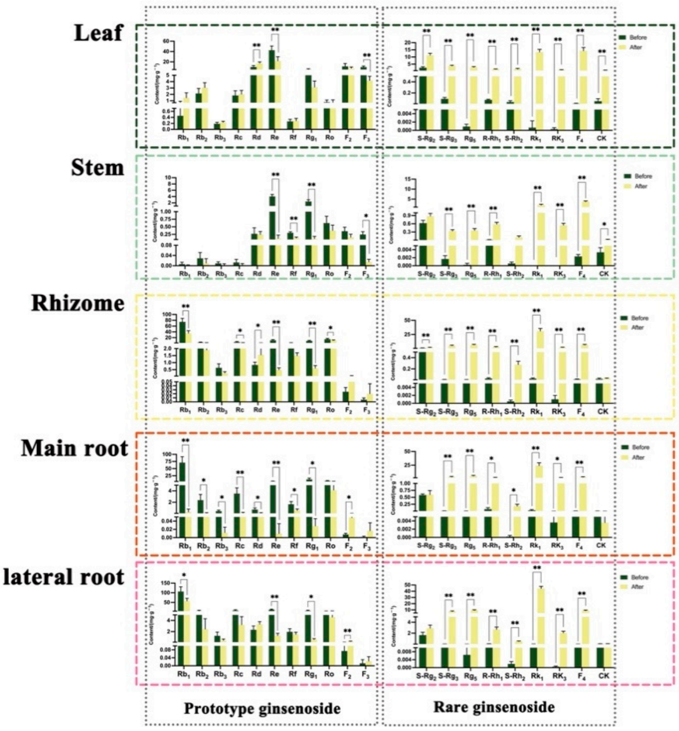

Figure S3 Comparison of ginsenoside content in five parts of 27-year forest ginseng before and after processing.

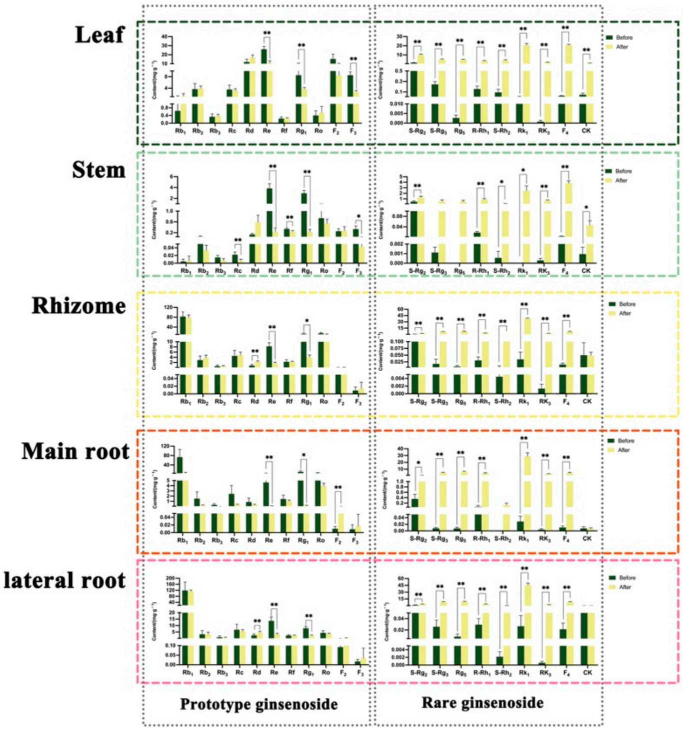

Figure S4 Cluster heat map of the content of five parts of ginseng.

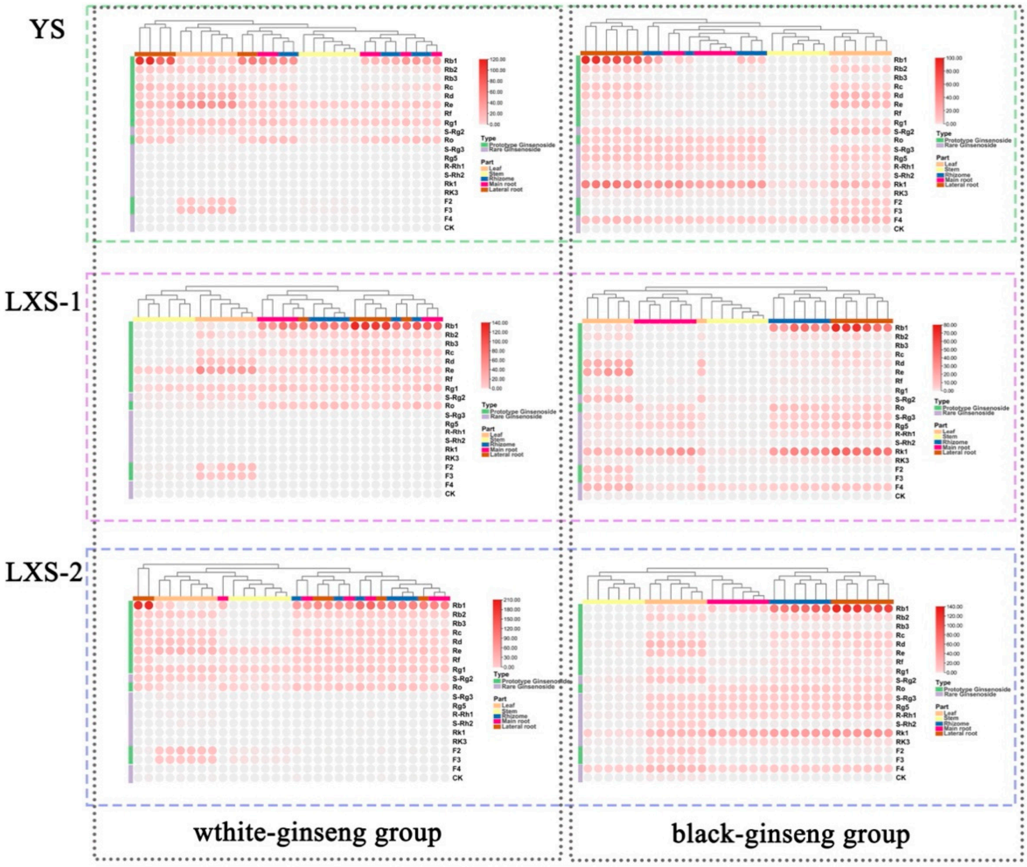

Figure S5 The PCA score plot.

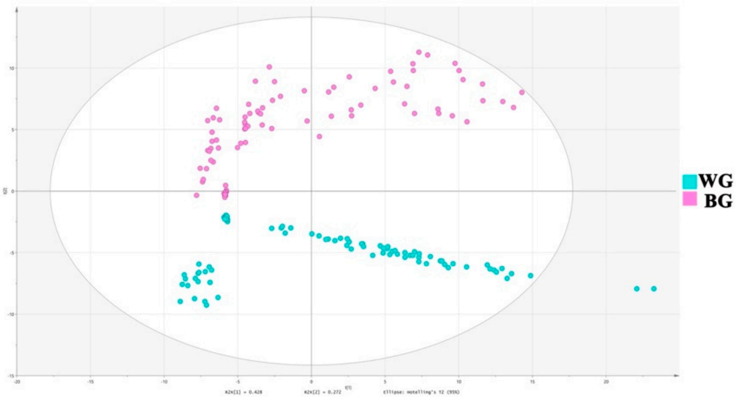

Figure S6 Cluster heat maps of four biomarkers of YS (A), LXS-1 (B) and LXS-2 (C).

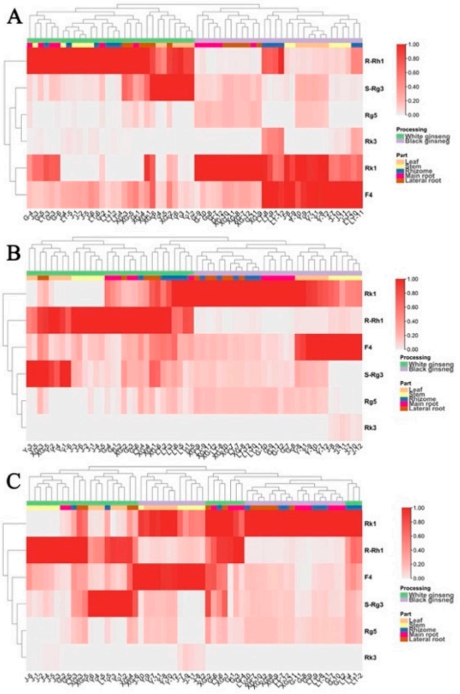

Figure S7 The PPI network analysis.

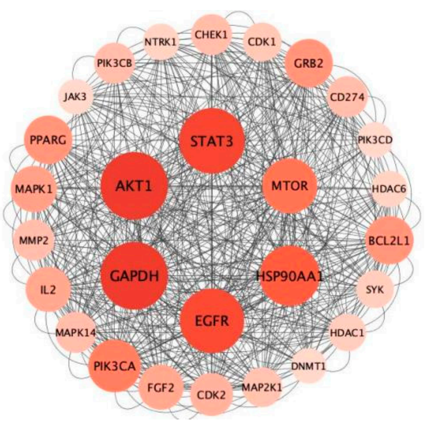

Supplement: Supplementary file 1 [file foods-13-02497-s001.zip › foods-3109837-supplementary.pdf]
